# Blended smartphone intervention for patients in opioid maintenance treatment in Iran: protocol for a randomized controlled trial

**DOI:** 10.1186/s12888-023-05007-0

**Published:** 2023-07-18

**Authors:** Nikolaos Boumparis, Alireza Noroozi, Eisa Naghizadeh, Andreas Meyer, Andreas Wenger, Afarin Rahimi-Movaghar, Michael P. Schaub

**Affiliations:** 1 Swiss Research Institute for Public Health and Addiction, University of Zurich, Konradstrasse 32, 8005 Zurich, Switzerland; 2 Iranian National Center for Addiction Studies, Tehran University of Medical Sciences, Tehran, Iran

**Keywords:** Opioid use disorder, Opioid maintenance therapy, Transdiagnostic treatment, Common mental health symptoms, Internet-based intervention, Blended treatment

## Abstract

**Background:**

The pattern of substance use in Iran is characterized by a high prevalence of opioid use and opioid use disorder (OUD). Although opioid maintenance therapy (OMT) has been introduced in Iran, approximately 50% of people with opioid use disorder remain unreached. Moreover, psychosocial treatment of OUD and common mental health symptoms during OMT is limited. Digital interventions have been shown to improve psychological distress, depression, anxiety, and post-traumatic stress disorder symptoms. In addition, providing psychoeducation and risk reduction counseling to prevent communicable diseases like HIV and infectious hepatitis is common via the Internet. However, despite these promising advances, no smartphone intervention in OMT has been investigated for the treatment of OUD and common comorbid mental health symptoms.

**Objective:**

We examine the effectiveness of adding a blended smartphone intervention based on community reinforcement approach, motivational interviewing- and cognitive behavioral therapy compared to OMT as usual that aims to improve OMT outcomes and addresses common mental health symptoms in OMT patients in Iran.

**Method:**

Adults with opioid dependence entering 8 treatment centers in Tehran, Iran will be randomly assigned to receive either OMT plus a smartphone intervention or OMT as usual. The primary outcomes will be the percentage of negative urine tests for illicit, non-prescribed use of opioids (opium, heroin, tramadol) and treatment retention. Secondary outcomes will include the longest period of abstinence from the illicit, non-prescribed use of opioids (opium, heroin, and tramadol) confirmed by urine samples, changes in communicable disease risk-taking behaviors, changes in stress and common mental health symptoms, and client satisfaction. Data analysis will follow the intention-to-treat principle and employ (generalized) linear mixed models.

**Discussion:**

This study will provide substantial knowledge for designing effective blended interventions for OUD. Moreover, it will investigate if treatment retention and OMT-related outcomes and common mental health symptoms can be improved by adding a smartphone intervention to OMT.

**Trial Registration:**

https://en.irct.ir/trial/53578.

**Supplementary Information:**

The online version contains supplementary material available at 10.1186/s12888-023-05007-0.

## Introduction

Iran has a high prevalence rate of opioid use. The 12-month prevalence of opioid use in the population aged between 15 and 64 is reported to be over 3.0%, and the 12-month prevalence of DSM-5 diagnosis of opioid use disorder (OUD) is 2.23% [1]. The gender gap in regard to opioid use in Iran is much larger compared to other countries [2, 3], which is likely due to socio-cultural factors [4, 5].

Over the past decades, consumption patterns have evolved from opium smoking – that still has a historical tradition – to heroin smoking and heroin injection, which has considerably increased the health consequences of opioid use in Iran [6, 7]. In fact, using non-sterile syringes is the most prevalent route (57.3%) of HIV transmission among all registered HIV-positive cases until the end of 2021 [8]. Furthermore, high prevalence rates for hepatitis C [7] and tuberculosis [9–11] have been reported among people who inject drugs in Iran.

Opioid maintenance therapy (OMT), using methadone and buprenorphine, was therefore introduced to respond to the HIV epidemic among those using opioids and scaled up rapidly [12, 13]. OMT programs are provided through a network of certified drug treatment centers and have shown promising effects for the treatment of OUD and for reducing the risk of HIV transmission [14–16]. However, there are limited resources to provide continuous psychosocial interventions necessary for the treatment of OUD and the management of common mental health symptoms, such as depression and anxiety, which are highly prevalent in patients with OUD [1].

Smartphone interventions for patients with OUD could help to close this gap by delivering psychological interventions with high fidelity while requiring fewer human resources and empowering individuals to self-manage mental health issues. The perceived anonymity, timeliness of access, and flexibility offered by digital interventions also evade commonly identified barriers to accessing care. Blended interventions that integrate face-to-face and digital interventions for patients with OUD could help to reduce this treatment gap. This study aims to develop a smartphone intervention and to deliver it in a blended treatment format for patients with OUD who enter OMT program and assess its effectiveness through a randomized controlled trial.

## Methods

### Study design

We will conduct a multi-center, two-arm, parallel-group randomized controlled trial (RCT) comparing an integrated community reinforcement approach- (CRA), motivational interviewing- (MI), and cognitive, behavioral treatment (CBT)-based smartphone intervention (called PROMPT for Promoting Recovery in Opioid Maintenance and Psychosocial Treatments) suitable for blended treatment in OMT with OMT as usual. Consequently, participants in the PROMT condition will receive access to the smartphone intervention in addition to OMT as usual; participants in the OMT as usual condition will receive OMT as usual but not access to PROMPT. The Ethics Committee of Tehran University of Medical Sciences (Code: IR.TUMS.MEDICINE.REC.1399.714) and the Ethics Committee of the philosophical faculty of the University of Zurich have approved the study protocol, informational letter, and informed consent process.

### Eligibility criteria

Patients will be eligible for the study if they meet the following criteria: (1) current diagnosis of opioid dependence (ICD-11), (2) receiving methadone or buprenorphine maintenance treatment for at least two weeks and not more than eight weeks, (3) ownership of a smartphone, (4) access to the Internet, (5) minimal age of 18 years, (6) provide written informed consent, (7) good command of and ability to read and write Persian. Exclusion criteria are (1) concurrent diagnosis of any other substance dependence (except tobacco) and (2) current severe or acute psychiatric disorders.

### Recruitment

The study will be advertised at drug treatment centers and other treatment settings where patients with opioid dependence are prevalent. Patients will be recruited at the INCAS clinic (https://enincas.tums.ac.ir), and seven further selected clinics in Tehran from patients recently admitted to OMT programs, including methadone and buprenorphine maintenance treatments. Patients interested in study participation will be informed about the study’s rationale, as well as: [1] study inclusion and exclusion criteria; [2] the two different study arms and their chance of being allocated into each one; [3] potential risks of participation; [4] safety arrangements during and after the study phase; [5] inability of the PROMPT intervention to replace face-to-face OMT; and [6] circumstances under which they should contact a professional at their treatment center. Moreover, they will be informed about their voluntary participation and their right to withdraw from the study at any time without consequences, except for the loss of further compensation. Written informed consent must be provided for study participation.

### Randomization

**Fig. 1:**
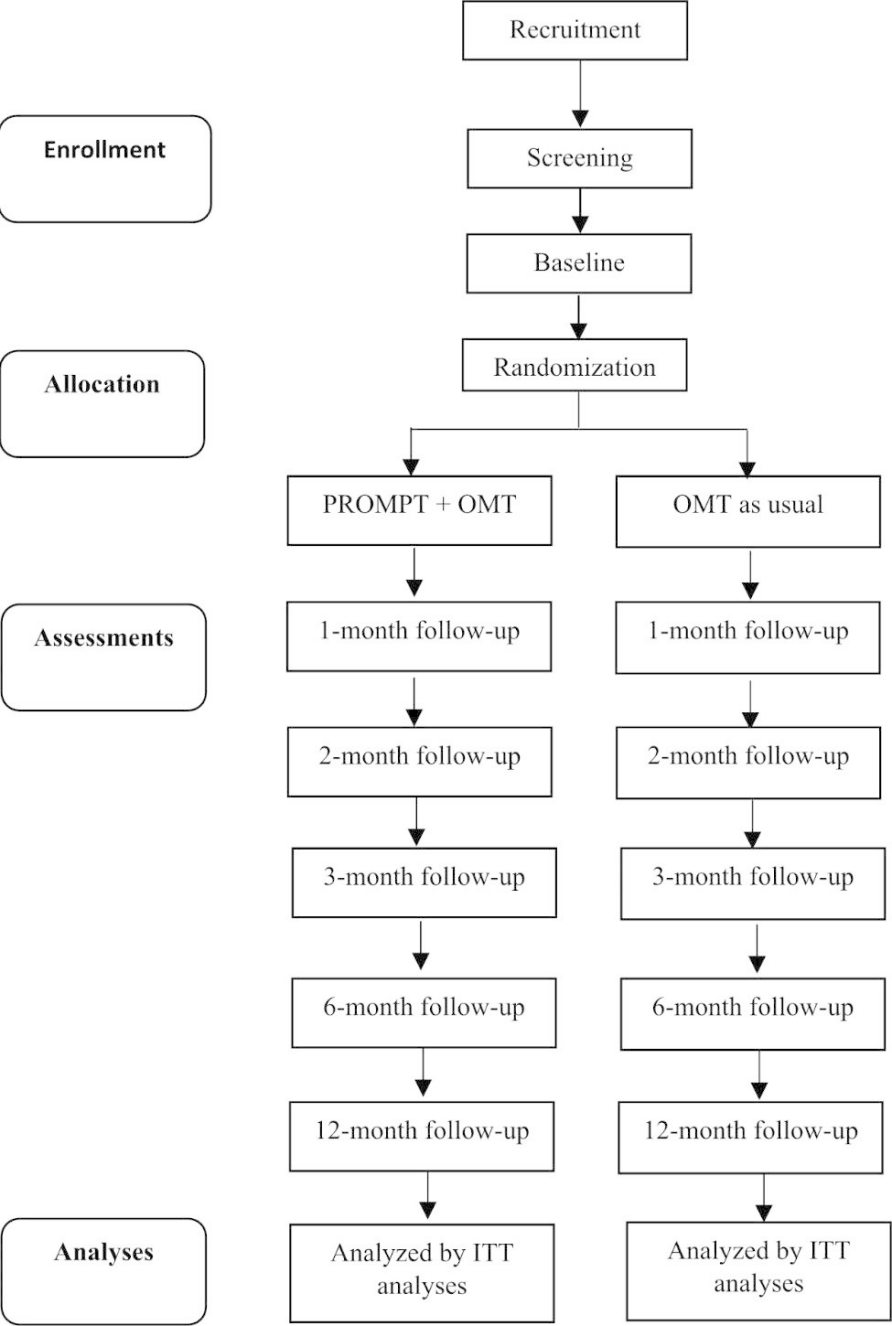
Flow diagram

After potential participants have provided informed consent, been deemed eligible according to inclusion and exclusion criteria, and have completed the baseline assessment, participants will be randomized by an automated computer program into either the treatment or control group in a 1:1 ratio. Completion of each follow-up assessment will result in participants being compensated with an equivalent of 5 CHF via an online voucher. Figure 1 provides an overview of the trial flow.

### Sample size

Based on the results of a systematic review and meta-analysis on internet interventions in adults with opioid dependence [17], we expect a Cohen`s d of about 0.35, comparing Study arm 1 (PROMPT) and Study arm 2 (OMT as usual). An overall sample size of N = 400 participants would have 80% power to detect the expected effect size when modeling a three-level generalized linear mixed model with six measurements (baseline, 1-, 2-, 3-, 6-, and 12-month follow-up) and eight treatment centers. Respective indices of baseline variance at the participant and treatment center level, as well as slope variance at the center level, and moderate heterogeneity in change over time, were based on a psychotherapeutic simulation study [18]. This calculation also considers an expected dropout rate of 30%, with a higher risk of dropout towards the end of the study. Calculations were performed using the package powerlmm in R [19].

### Opioid maintenance therapy as usual

Certified opioid agonist treatment centers provide OMT services through a multidisciplinary team consisting of a trained general physician, clinical psychologist, and nurse. Physician visits, counseling sessions, and the dispensing of opioid agonist medications are all provided at the centers. Each multidisciplinary team is allowed to provide treatment for a maximum of 230 OMT patients, including 100 patients on methadone, 80 receiving buprenorphine, and 50 receiving opium tincture.

The type of treatment regime (i.e., assisted withdrawal, maintenance treatment) and agonist medication (methadone or buprenorphine) are chosen by the professionals based on patients’ substance use history and previous experiences with opioid pharmacotherapies and preferences. General guidelines exist for opioid agonist induction, which the physician tailors to patients’ needs. There is no upper or lower dose limit for methadone during the maintenance phase, although methadone doses ranging from 80 to 120 mg/day are generally considered optimal. Due to buprenorphine’s “ceiling effect” in doses above 32 mg/day, physicians may prescribe up to 32 mg of buprenorphine daily. However, it has been demonstrated that daily doses exceeding 24 mg yield minimal clinical benefits. Consequently, physicians are advised to reevaluate the patient’s condition and contemplate alternative therapeutic approaches if stabilization cannot be attained at this dosage level.

During treatment, patients are regularly visited by a general physician and participate in weekly individual counseling sessions with the center’s clinical psychologists for the first 12 weeks of treatment. Morphine and methamphetamine urine tests are obtained routinely to monitor concurrent illicit substance use. At the beginning of OMT, patients must regularly come to the center and consume their dose under professional supervision. Contingent to the behavioral stabilization (i.e., regular attendance and negative urine tests), take-home doses start and may increase gradually to up to two weeks from the start of their fourth month of treatment [12, 20].

### Blended PROMPT intervention

Blended interventions represent a relatively new approach in psychological treatments, integrating the flexibility and accessibility of digital tools with the personalized care and guidance provided by face-to-face therapy. Based on the integrated blended treatment manual, where psychologists blend the digital component with their face-to-face treatment. Blended interventions allow patients to benefit from a solution that retains the advantages of online interventions - enhanced patient self-management, cost-effectiveness and efficient use of therapist time - while allowing close monitoring and guidance by therapists. The PROMPT intervention facilitates the recording and tracking of patient progress, including tasks such as monitoring daily drug use and cravings, which will be systematically incorporated into counseling sessions.

PROMPT is a web-based intervention developed by the Swiss Research Institute for Public Health and Addiction (ISGF) and the Iranian National Center for Addiction Studies (INCAS) that was designed to be delivered as a blended smartphone intervention in addition to OMT services in Iran. PROMPT consists of a dashboard, a consumption diary, a craving tracker, and 10 modules designed to improve OMT outcomes based on the principles of MI, CRA, and CBT. Participants allocated to the blended intervention condition will receive, in addition to OMT as usual, access to PROMPT. The smartphone intervention contains modules based on psychoeducation, risk-reduction counseling, self-monitoring and visual change tracker feedback, integrated CRA, MI, and CBT. A detailed overview of the included modules can be found in Table 1.

**Table 1:** Content of the intervention

Module	Content	Therapeutic Approach	Reasoning
Module 1: Introduction	-General overview -Introduction to the diary -Introduction to the craving tracker		Gradual introduction and preparing the patient to start the therapeutic modules.
Module 2 : Motives for change	-Pros and cons of drug use, including risks of communicable disease transmission; clarification of confidence in change	Motivational interviewing techniques [32]	A targeted increase in motivation.
Module 3: Craving and lapses	-Concept of cravings -Ways to deal with cravings -Strengthening positive experiences unrelated to substance use -Introduction and planning of daily mindfulness-based meditation exercise -Relapse prevention -Dealing with lapses/relapses -Strengthening refusal skills for use in high-risk situations	CBT [33], Community Reinforcement Approach [34], including mindfulness based meditation [35]. CBT for relapse prevention [36]	Learning to understand the cravings concept and how to handle cravings. Introduction to mindfulness exercises and creation of an exercise plan. To avoid relapses resulting in cessation of the intervention or OMT. Strengthening refusal skills for use in high-risk situations.
Module 4: Triggers	-Identifying personal high-risk situations -Recognizing seemingly irrelevant, but triggering decisions.	CBT for relapse prevention [36]	Explanation of the CBT concept of risky situations and their linkages, as well as how such situations can be avoided.
Module 5: Harm reduction	- Psychoeducation on harm reduction -Overdose prevention -Reducing high-risk sexual and substance use behaviors	Harm reduction principles [37, 38]	Psychoeducative prevention of overdose and the transmission of blood-borne viruses.
Module 6: Working on needs I	-Strengthening social contacts and positive experiences unrelated to substance use -Introduction of the activation diary -Decreasing excessive ruminations -Developing healthier sleeping habits	Community Reinforcement Approach [34], behavioral activation approach [39], excessive rumination and healthier sleep exercises [40]	To increase the number of non-drug-related enhancers and, thus, prevent relapses. To improve the handling of potentially emerging, ego-threatening thoughts and sleep problems that may arise during the introduction of OMT.
Module 7: Working on needs II	-Strengthening social contacts and positive experiences unrelated to substance use (continuation) -Introduction and maintenance of activation diary -Relationships between substance use, problems, and depressive symptoms	Social problem solving Approach [41]	To promote an understanding of the potential emergence of depressive symptoms associated with the introduction of OMT and the ego-threatening thoughts and life problems that may arise.
Module 8: Address problems	-Skills to deal with solvable and unsolvable problems	Social problem solving Approach [41]	To promote an understanding of the potential emergence of depressive symptoms associated with the introduction of OMT and the ego-threatening thoughts and life problems that may arise.
Module 9: Negative thoughts	-Inner thoughts and how they influence mood and drug-taking behaviors -Common thinking errors -Challenging automatic negative thoughts	CBT approach of our previous Internet-based study [42]	CBT-based combating of negative thoughts arising from the introduction of OMT and the negative thoughts associated with it.
Module 10: Preserve sucess	-Review of the program -List of 5 personalized points to help secure achievements	Community Reinforcement Approach [34]	Appreciation of what has been achieved; repetition and consolidation of what has been learned to increase the likelihood of long-term success.

### Dashboard

**Fig. 2:**
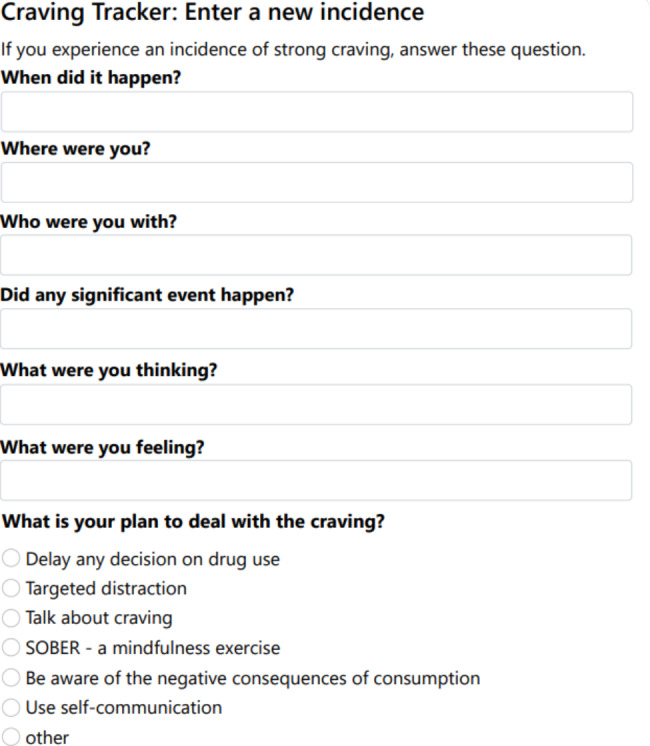
Craving tracker

The dashboard provides a convenient and easy-to-read overview of important information. Participants can view their program start date and remaining days, scheduled follow-up assessments and their completion status, and continue the intervention modules where they left off. Additionally, participants have the opportunity to input their substance use and craving (Fig. 2) in a table and see their past use visualized in a graph.

### Intervention modules

There are ten self-help intervention modules. They are depicted on an overview webpage linked via the main menu. The user’s progress for each module is also listed on the dashboard. The anticipated duration of the smartphone intervention is three months; during this time, all modules are discussed with the patient’s psychologist. Patients are advised to work through the modules in their presented order unless there is an urgent need for a user to skip to a specific module. The psychologists will recommend repeating the modules that are perceived as beneficial. A progress bar in the module overview shows how far a participant has progressed in each module, turning fully green once the entire module has been completed.

### Diary

**Fig. 3:**
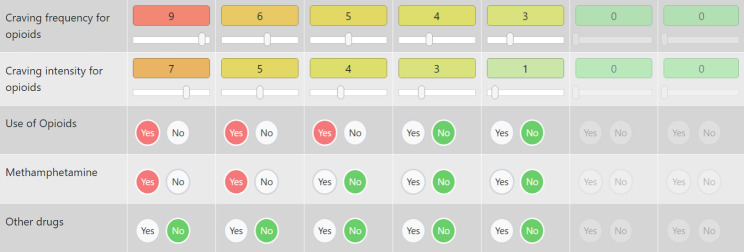
Consumption diary

**Fig. 4:**
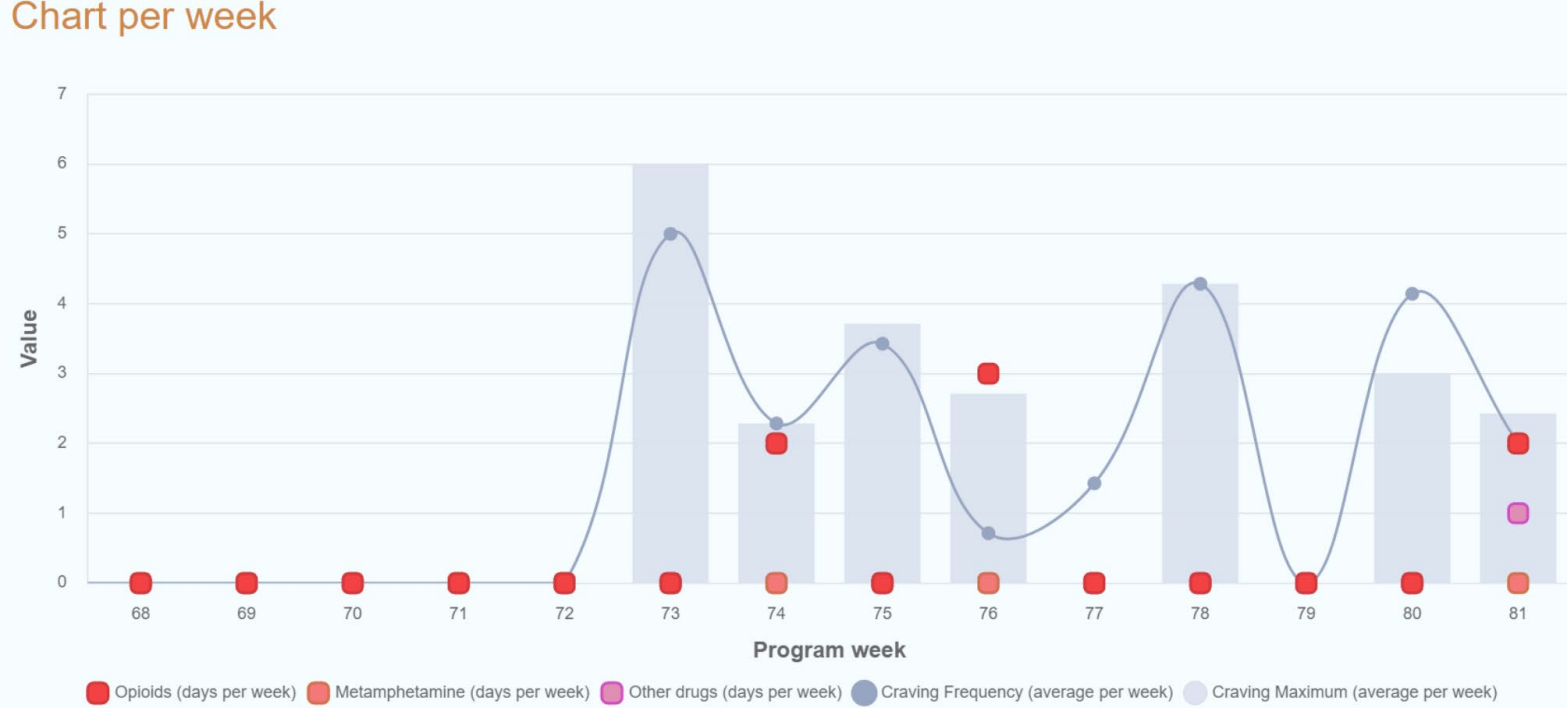
Visual feedback resulting from the consumption diary

The diary is a central monitoring instrument for participants. In two tables each with 7 days representing the current and the previous week, they can enter their craving frequency and intensity for opioids on a scale from 0 to 10 and whether they used opioids, methamphetamine or other substances via three yes-no-switches. This information is visualized in two graphs, a chart per day for shorter periods and a chart per week for long term progress (Figs. 3 and 4).

### Other elements

PROMPT includes a section with general information related to risks associated with opioid use and an introduction of OMTs as standard treatment of opioid dependence. Additionally, some modules require participants to complete exercises by selecting checkboxes or providing written responses.

### Technical specifications

PROMPT is built on the Drupal 9 content management system and uses a relational database. It will be managed by an IT developer at the institution where the principal investigator works. Access to the program is protected by encrypted and password-secured connections. Participants only have access to their own data, while the administrator has full access to all data entered by all participants. Data is entered online by the users themselves, and the system includes built-in validation to ensure accurate data, such as accepting only numbers for certain fields and requiring certain fields to be filled out.

With the assistance of the treatment center’s psychologist, participants will register for the program by choosing a unique username and a secure, personal password to safeguard against unauthorized access. Data is stored on a web space hosted by an external provider that meets Swiss IT security regulations, and employees of the web host are required to provide biometric identification to access the infrastructure. Data is extracted from the web-based database using Drupal and PHPmyAdmin and stored locally at the PI’s institution for processing and archiving. The investigator ensures that all participants’ right to privacy is upheld and that all personnel involved in the study comply with applicable privacy laws. No individual data will be published or presented at scientific meetings.

### Training of the treatment team

The therapists involved in the study are trained in OMT and have extensive experience working in certified opioid agonist treatment centers in Iran. They will be working with participants from both treatment arms of the study after receiving additional training on integrating the PROMPT treatment protocol into their routine OMT practice. To ensure fidelity to PROMPT intervention, psychologists will also participate in weekly group supervision meetings in the first months of recruitment, followed by biweekly meetings led by our research team. The objectives of supervisory meetings are threefold: to foster the advancement of psychologists’ expertise and clinical competencies in utilizing PROMPT, to guarantee consistent implementation for participants in the intervention group, and to offer the necessary support.

### Assessments

#### Primary and secondary outcomes

The primary outcomes will be the percentage of negative urine tests for illicit, non-prescribed use of opioids (opium, heroin, tramadol) between each measurement point (baseline, 1-, 2-, 3-, 6-, and 12-month follow-up) and treatment retention indicated by weekly percentage of participants who continue participation defined as receiving planned services (i.e., opioid agonist medication, physician’s visits, counselling sessions) and completing the study assessments. Secondary outcomes will be the longest period of abstinence for illicit, non-prescribed use of opioids (opium, heroin, tramadol) confirmed by urine samples; the number of self-reported other-street-drug free days over the preceding 7 days (Timeline Follow Back method) [TLFB], [21]; the number of self-reported opioid-free days over the preceding 7 days [TLFB]; the number of missed OMT appointments; screening of methamphetamine use via monthly urine tests; identification of benzodiazepine use through monthly urine screenings; detection of THC presence via monthly urine analyses; changes in communicable disease risk-taking behaviors (Blood-borne Virus Transmission Risk Assessment Questionnaire) [BBV-TRAQ], [22]; HIV test results; symptoms of stress (Perceived Stress Scale) [PSS-10], [23] and other common mental health symptoms, such as depression (Patient Health Questionnaire) [PHQ-9], [24], anxiety (Generalized Anxiety Disorder Screener) [GAD-7], [25]; PTSD (International Trauma Questionnaire for PTSD) [ITQ] [26]; adult attention-deficit/hyperactivity disorder symptoms interview [NSC-R]; alcohol use disorder symptoms (Alcohol Use Disorders Identification Test) [AUDIT] [27]; nicotine dependence (Fagerstrom Test for Nicotine Dependence) [FTND] [28], and, client satisfaction (Client Satisfaction Questionnaire [CSQ-8], [29]. Assessments will be conducted by the participating psychologists, who will assist the patients with the input of the relevant data in the web-based questionnaires.

Furthermore, any negative effects due to the smartphone intervention will be measured via the Negative Effects Questionnaire [30] at all follow-up assessments. As an indicator of treatment adherence, data will be collected on how many modules have been completed and how many OMT physicians’ visits and counseling sessions are attended by each participant. The primary and secondary outcomes and the exact time points at which each measure will be applied are presented in Table 2.

**Table 2:** Overview of measures and their time of assessment

	Baseline	Month 1	Month 2	Month 3	Month 6	Month 12
Sociodemographic characteristics	x					
Addiction treatment history	x					
Percentage of negative urine tests (illicit, non-prescribed opioids)		xxxx	xxxx	xxxx	x	x
The longest period of abstinence from the illicit opioids		x	x	x	x	x
Number of missed OMT appointments		x	x	x	x	x
Blood-borne Virus Transmission Risk Assessment Questionnaire (BBV-TRAQ)	x			x	x	x
Number of self-reported opioid use/free days - (TLFB)	x	x	x	x	x	x
Self-reported other street-drug use/free days - (TLFB)	x	x	x	x	x	x
Perceived Stress Scale (PSS-10)	x			x	x	x
Patient Health Questionnaire for Depression (PHQ-9)	x			x	x	x
Generalized Anxiety Disorder Screener (GAD-7)	x			x	x	x
International Trauma Questionnaire for PTSD (ITQ)	x			x	x	x
Client Satisfaction Questionnaire (CSQ-8)		x	x	x	x	x
Negative Effects Questionnaire		x	x	x	x	x
Methamphetamine (MA) urine test (monthly)	x	x	x	x	x	x
Benzodiazepine urine test (monthly)	x	x	x	x	x	x
THC urine test (monthly)	x	x	x	x	x	x
HIV test result	x			x	x	x
Adult ADHD Interview (NSC-R)	x					
Alcohol Use Disorder Identification Test (AUDIT)	x			x	x	x
Fagerstrom Test for Nicotine Dependence (FTND)	x			x	x	x

### Statistical analyses

Data will be analyzed according to the intention-to-treat principle (ITT). We will examine for differences in all primary and secondary outcome variables between the two study arms at baseline and follow-up assessments using (generalized) linear mixed models. We will specifically model repeated measures nested within participants and participants nested within treatment centers by defining respective random intercepts and slopes. Study conditions and time will be modeled as fixed effects. Appropriate distributions for non-normal continuous and/or count outcomes will be specified (e.g., negative binomial, zero-inflated, Poisson). Subject to the sample’s actual gender distribution, we will investigate the intervention’s gender-specific effects by including a gender*treatment*time interaction term. If this is not possible due to inadequate sample size (i.e., the model is not identifiable), separate subgroup analyses will be run, which will allow the determination of differential treatment effects.

## Discussion

This study aims to investigate the effectiveness of blended OMT treatment in which OMT as usual, is delivered in combination with a smartphone intervention for patients with opioid dependence in Iran.

The prevalence and burden of OUD in Iran, both on an individual and societal level, warrant a variety of effective treatment strategies. So far, research on the effectiveness of digital interventions for the treatment of OUD is primarily limited to add-on interventions that have been investigated in the United States [31]. However, the scientific evidence on the value of, in particular, blended interventions in developing countries for the treatment of OUD is scarce.

Through the integrated approach of blended interventions, we expect to provide our participants with both the benefits of digital interventions and the advantages of face-to-face therapy. However, there are many unknowns regarding blended interventions, such as how to combine these treatment modalities and the characteristics of therapist support. Our study will attempt to provide relevant answers to these questions. Furthermore, we will better understand to what extent the blended intervention suits patients in OMT settings by exploring treatment adherence, dropout, and satisfaction with the blended intervention. In addition, by monitoring the activities of our patients in the smartphone intervention (e.g., time spent in each module), we will be able to get insights into what happens during the treatment period. This will further increase our knowledge of the suitability of blended treatments for patients in OMT.

We will not interfere with the current practices of OMT as usual. By permitting the practice of usual care for OMT patients, the generalizability of our results is enhanced. Given the heterogeneity of OMT as usual, this could also be regarded as a limitation. However, since our study is designed to be a pragmatic trial, we aim to compare the effectiveness of the blended intervention with the current OMT as usual practice. Therefore, OMT as usual will be monitored carefully to control for potential confounding effects.

This study will provide substantial knowledge to aid in developing effective blended interventions delivered in OMT for Persian-speaking populations. In addition, to aid the implementation and ensure treatment fidelity, therapists will receive training on how to deliver a blended intervention and technical support from the research team. Usage will be facilitated via the implementation of open-source software that can be used and maintained with few resources. Thus, long-term sustainability will be achieved.

## Electronic supplementary material

Below is the link to the electronic supplementary material.

Supplementary Material 1: SPIRIT 2013 Checklist

## Data Availability

Not applicable.
